# Reproducibility and Transparency by Design*

**DOI:** 10.1074/mcp.IP119.001567

**Published:** 2019-07-04

**Authors:** Vladislav A. Petyuk, Laurent Gatto, Samuel H. Payne

**Affiliations:** ‡Pacific Northwest National Laboratory, Richland, WA; §de Duve Institute, Université Catholique de Louvain, Brussels, Belgium; ¶Brigham Young University, Provo UT

**Keywords:** Bioinformatics, Data Evaluation, Data Standards, Computational Biology, Algorithms, Open Science, Reproducibility, Transparency

## Abstract

The reproducibility of bioinformatics analyses can be elevated to equal status with biological discovery. To achieve this, reproducibility must become part of the process, not an afterthought.

## Graphical Abstract

**Figure:**
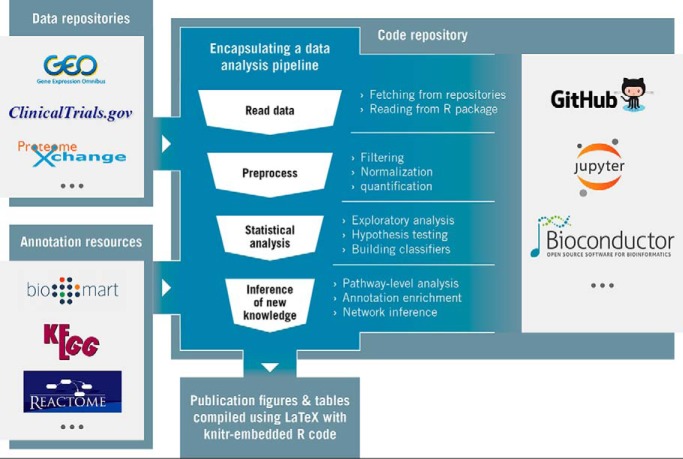

**Highlights**

- Repositories are enabling sharing of data.
- Sharing analyses remains a major stumbling block for reproducibility.
- Three simple steps can enable sharing analyses, with nominal effort.

To truly achieve reproducible research, having reproducible analytics must be a principal research goal. Biological discovery is not the only deliverable; reproducibility is an essential part of our research.

Public trust of scientific research is affected by the clarity of published conclusions and also the perceived transparency of the method. Although irreproducibility is not exclusive to biology, strong public interest in environmental and biomedical discoveries seems to have focused the spotlight here following a number of high-profile studies that failed to be reproduced (1–6). In this report, we specifically focus on the linked issues of reproducibility and transparency of integration and analyses for multi-omics data. Unlike data generation where biological variability is expected to be manifest, computational analyses should be completely and exactly reproducible. Unfortunately, the documentation of data processing, analysis, and statistical algorithms in publications is usually not sufficiently detailed. This lack of detail is especially problematic for multi-omics characterizations where the complex statistical integration is essential to merging disparate data types (*e.g.* clinical, proteomics, genomics, etc.).

## 

### 

#### Making Reproducibility a Priority. Where Are the Gaps?

There are many stages of a multi-omics project, and recent efforts have made significant improvement on transparency of data files and selected steps of analysis. *MCP* and other journals have been leaders in requiring the complete sharing of raw data and preliminary processing (7–9). In a multi-omics project, it is now common to require that the mass spectrometry instrument files are freely shared via public repositories, which exist for genomics (10), proteomics (11), and metabolomics (12). Spectral identification must also be reported with the software and associated parameters. Pipelines run through the BioContainers (13) facilitate this recording. Although some popular tools with a graphical interface may not currently store this workflow meta-data, we feel that this is rapidly becoming a demand of both the users and publishers.

After obtaining quantitative molecular data, there is still a lot of work before publication. This includes merging genomic and proteomic data tables, binning samples into phenotypic groups based on multi-omic clustering, functional enrichment analysis, metabolic network modeling, and so on. Unfortunately, the current efforts to mandate data sharing have focus on just the data. Data interpretation and statistical analyses that support scientific conclusions are an equally essential component of our work and must also be openly shared. We write this commentary to highlight the need for greater efforts in the open sharing of analyses.

Although it is a narrow topic, we feel it is important to discuss. As mandated data sharing resolves a portion of the overall transparency/reproducibility challenge, the unaddressed issue remains the sharing of analyses. Moreover, our solution is not that difficult to implement for the new generation of data savvy researchers. It does not require large grants to fund computational/storage infrastructure; it can be done by individual researchers with a modicum of effort. Thus, without delay, journals can start to encourage or enforce the open sharing of computational and statistical data interpretation.

As its central feature, our solution encapsulates the entire data analysis in software, including the creation of publication quality figures. We want to make it easy for peers to do exactly the same analyses in a publication—specifically the critical final steps where data interpretation happens. For example, when discussing an assertion in the results section, it is common to parenthetically list the *p* value and a specific test. To increase the transparency and reproducibility of this assertion, we should share the actual software code that produced this *p* value. Multiple modern software platforms have made this level of transparency achievable with modest effort, including Jupyter notebooks and R markdown (14, 15). Our support for these technologies is not meant to be exclusive but merely convenient as many publications already utilize Python and R/Bioconductor (16). We strongly advocate for the following three steps: code for analysis and figures posted to an open version control software repository like GitHub (17), data tables used by the analysis be posted in the same repository or linked to a password-free download if too large, and the URL to specific scripts in a repository be prominently listed in figure legends and methods sections. The effect of these three would be that anyone interested in a specific figure or conclusion of the paper could easily find the exact analysis method and fully repeat the computation. Indeed, this approach for reproducibility has already been used in a few exemplary publications (18–21).

#### Looking Forward

The benefits of true transparency have been previously noted (22, 23), and we reiterate that our proposed solution has lasting positive effects for the principal investigator, funding agencies, peer review, collaborators, and the general public. The solution is flexible and applicable to the broad needs of multi-omics integration for climate research, clinical proteogenomics, systems biology, computational neuroscience, and so on.

As multi-omics measurements continue to revolutionize environmental and biomedical research, biology more explicitly becomes a data science. Most graduate programs now require statistics courses, where students learn tools like R and Python. Given the enormous societal impact that comes from scientific discoveries, the transparency of our data and methodology is a critical component of the scientific venture. As large data repositories have begun to capture much of the raw data generated for experiments, we have suggested a companion method to disseminate and expose data analysis methods. Ultimately, the transparency of full disclosure will expose any actual problems underlying irreproducibility in a manner where other researchers can help to correct and advance science.
